# Brain Abscess Following CPX‐351 Treatment in a Patient With Acute Myeloid Leukemia

**DOI:** 10.1155/crh/3668400

**Published:** 2026-05-21

**Authors:** Yusuke Yamaga, Shoichiro Okazaki, Yuri Sakuma, Daimon Siraisi, Takahiro Nishiyama

**Affiliations:** ^1^ Division of Hematology, Ichinomiya Municipal Hospital, Ichinomiya, Aichi, Japan, municipal-hospital.ichinomiya.aichi.jp; ^2^ Department of Neurosurgery, Ichinomiya Municipal Hospital, Ichinomiya, Aichi, Japan, municipal-hospital.ichinomiya.aichi.jp

**Keywords:** acute myeloid leukemia, *Bacillus cereus*, brain abscess, CPX-351, immunocompromised patients

## Abstract

We describe a rare case of a brain abscess occurring during CPX‐351 induction therapy for acute myeloid leukemia (AML). The patient experienced recurrent febrile neutropenia during the myelosuppression period and developed left‐sided spatial neglect during hematologic recovery. Brain magnetic resonance imaging revealed a solitary abscess in the right parietal lobe. Burr hole drainage yielded *Bacillus* species, and subsequent treatment with vancomycin and linezolid, followed by endoscopic abscess resection, led to gradual neurological recovery. Complete remission of AML was achieved, and consolidation therapy with venetoclax plus azacitidine was initiated. CPX‐351 is associated with prolonged myelosuppression and relatively low gastrointestinal toxicity, which may reduce the risk of Gram‐negative sepsis but increase susceptibility to Gram‐positive infections, such as catheter‐related bacteremia. Clinicians should consider the possibility of a brain abscess in patients presenting with neurological symptoms during AML treatment, as early diagnosis and prompt surgical and antimicrobial management are critical for favorable outcomes.

## 1. Introduction

CPX‐351, a liposomal formulation of daunorubicin and cytarabine, has emerged as an important induction regimen for acute myeloid leukemia (AML) with myelodysplasia‐related changes (AML–MRC), particularly among older adults and patients with therapy‐related AML [1]. Compared with conventional intensive chemotherapy, CPX‐351 is associated with lower gastrointestinal toxicity, potentially reducing the risk of Gram‐negative bacteremia secondary to mucosal barrier injury [2]. However, it induces prolonged and profound myelosuppression, increasing susceptibility to severe infections, particularly those caused by Gram‐positive organisms, such as catheter‐related bloodstream infections (CRBSIs) [3].

Central nervous system (CNS) infections during AML treatment are uncommon, and brain abscesses are particularly rare and challenging to diagnose or manage. Reported pathogens include *Bacillus cereus* [4–6], fungal species such as *Aspergillus* and *Mucorales* [7, 8], and, more rarely, *Acanthamoeba* [9]. These infections often lead to considerable neurological deficits and can be fatal. Although advances in antimicrobial therapy and surgical techniques have improved survival in some cases, early diagnosis and prompt intervention remain crucial [10].

Here, we report a rare case of a brain abscess caused by *Bacillus* species in a patient receiving CPX‐351 induction therapy for AML–MRC. This case underscores the importance of early recognition and individualized management of CNS infections due to atypical pathogens during profound immunosuppression.

## 2. Case Presentation

A 60‐year‐old Japanese man was evaluated for pancytopenia. His medical history included Type 2 diabetes mellitus, myocardial infarction treated with percutaneous coronary intervention, abdominal aortic aneurysm, and resolved hepatitis B infection. His regular medications included magnesium oxide, carvedilol, rabeprazole, clopidogrel, linaclotide, and telmisartan. He had no known drug allergies and had smoked 20 cigarettes daily from age 20–42 years but had since quit.

**FIGURE 1:**
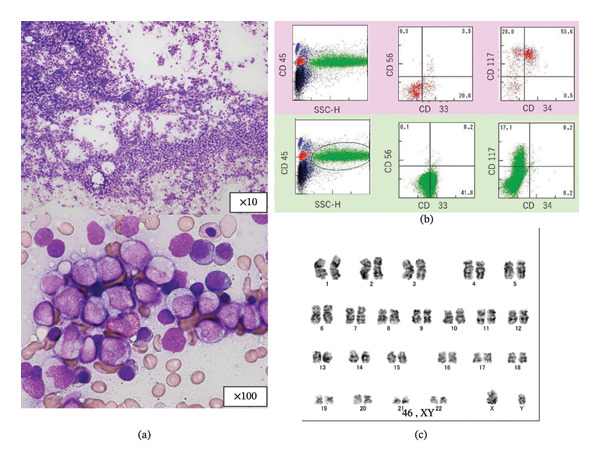
(a, b) Bone marrow smear showing hypercellularity (May–Grünwald–Giemsa stain, × 10). Higher magnification (× 100) reveals myeloblasts negative for CD34 and positive for CD117 (c‐Kit) by flow cytometry. (c) Karyotypic analysis showing 46, XY (20/20 cells).

Laboratory tests showed a white blood cell count (WBC) of 900/µL, absolute neutrophil count (ANC) of 153/µL, hemoglobin level of 6.7 g/dL, platelet count of 57,000/µL, and C‐reactive protein level of 0.03 mg/dL. Bone marrow aspiration revealed hypercellular marrow with dysplasia in all three lineages and myeloblasts positive for myeloperoxidase (MPO), negative for CD34, and positive for c‐Kit—findings consistent with AML–MRC (Figure 1). The patient was admitted and started on induction chemotherapy with CPX‐351 (Day 1). On Day 14, he developed febrile neutropenia (FN), and cefepime therapy was initiated. *Staphylococcus epidermidis* was isolated from catheter‐drawn blood cultures earlier than from peripheral samples, indicating a CRBSI. The central venous catheter was removed, and vancomycin was added but discontinued on Day 17 based on susceptibility results.

On Day 29, FN recurred with radiological evidence of pneumonia. Meropenem, liposomal amphotericin B (L‐AMB), and granulocyte colony‐stimulating factor (G‐CSF) were initiated. By Day 38, hematologic recovery was noted (WBC 3200/µL, ANC 2336/µL, hemoglobin 9.9 g/dL, and platelets 76,000/µL). However, the patient developed new‐onset left hemispatial neglect. Brain MRI revealed a solitary abscess measuring 30 × 50 × 43 mm in the right parietal lobe. The behavioral inattention test (BIT) score was 60/146. Burr hole drainage was performed the same day, and high‐dose meropenem in combination with vancomycin was administered. Considering CNS penetration, antifungal therapy was switched from L‐AMB to voriconazole. Culture of the abscess fluid yielded *Bacillus* species, and vancomycin administration was continued (Table 1).

**TABLE 1:** Summary of the patient’s clinical course, causative pathogens, and antibiotic susceptibility.

	**CRBSI**	**Pneumonia**	**Brain abscess**

Onset of infection	Day 15	Day 29	Day 38
Causative bacteria	*Staphylococcus* epidermidis	Normal flora	*Bacillus* species
Antibiotic susceptibility	Sensitive to all antibiotics	—	Unable to identify

**FIGURE 2:**
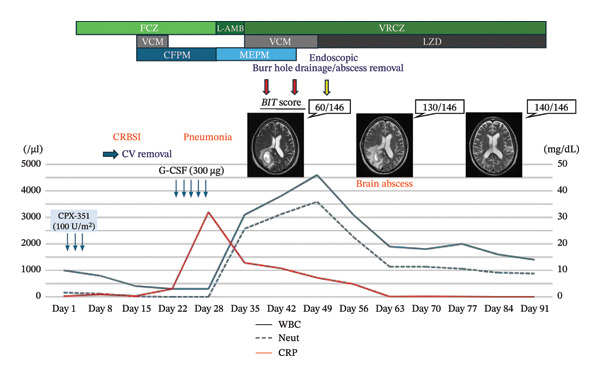
Timeline of the clinical course. BIT, behavioral inattention test; FCZ, fluconazole; L‐AMB, liposomal amphotericin B; VRCZ, voriconazole; CFPM, cefepime; MEPM, meropenem; VCM, vancomycin; LZD, linezolid.

On Day 45, a second burr hole drainage was performed because of a persistent abscess. As the lesion showed no improvement, vancomycin was replaced with oral linezolid on Day 51. Endoscopic abscess removal was performed on Day 52, resulting in gradual reduction in abscess size. Neurological symptoms improved progressively after initiation of linezolid therapy, with BIT scores increasing to 130/146 on Day 63 and 140/146 on Day 84. Linezolid therapy was completed on Day 93, and the patient was subsequently discharged (Figure 2).

The patient achieved complete remission of AML following CPX‐351 induction. WT1 mRNA levels in peripheral blood, which had been 4.3 × 10^3^ copies/µg RNA at baseline, became undetectable. After confirming radiological resolution of the brain abscess, consolidation therapy with venetoclax plus azacitidine was initiated.

## 3. Discussion

Brain abscess is a rare but potentially fatal complication in patients with AML, particularly during periods of profound immunosuppression caused by intensive chemotherapy. Since the 2000s, only a few cases have been reported (Table 2). Among the identified pathogens, *Bacillus cereus* is relatively common, typically emerging during the neutropenic phase and often leading to rapid clinical deterioration [4–6]. Although many cases have been fatal, some patients have survived with aggressive multimodal treatment, including surgical drainage and the administration of CNS‐penetrating antibiotics such as linezolid [6].

**TABLE 2:** Reported cases of brain abscess during AML treatment since 2000, including pathogens, presumed routes of infection, therapeutic strategies, surgical interventions, and clinical outcomes.

Ref	Year	Age/sex	Underlying AML	Pathogen	Route of infection	Therapy	Surgery	Outcome
Sakai et al. [4]	2001	67/F	ALL	*B. cereus*	Intestinal tract	VCM + LVFX	Burr hole	Survived
Kuwabara et al. [5]	2006	54/F	AML	*B. cereus*	Catheter‐related	MEPM + VCM	None	Survived
Koizumi et al. [6]	2020	54/F	AML	*B. cereus*	Not clear	MEPM + VCM + LZD	None	Survived
Shariati et al. [7]	2021	Various	AML/post‐HSCT	*Aspergillus*	Sinus/lung	AMPH‐B, VRCZ	Mixed	Mixed
da Silveira et al. [8]	2024	56/M	AML	*Mucorales*	GI dissemination	AMPH‐B	None	Died
Nampoothiri et al. [9]	2018	19/M	AML	*Acanthamoeba*	Not clear	ST + RFP + AZM	Surgery	Survived
Cavazza et al. [10]	2024	48/F	AML	*Not specified*	Lung	MEPM + ISCZ + PSL	Multisurgery	Survived
This case	2025	60/M	AML‐MRC (CPX‐351)	*Bacillus spp.*	Catheter‐related	VCM → LZD + VRCZ	Burr hole + endoscopy	Survived

*Note:* Most reported cases occurred during induction therapy or profound neutropenia, emphasizing this period as a high‐risk phase for brain abscess in patients with AML.

Fungal brain abscesses, particularly those caused by *Aspergillus* species, also present major therapeutic challenges in AML patients, and voriconazole has demonstrated efficacy in selected cases [7]. Infections due to *Mucorales* or *Acanthamoeba* are rare but are typically associated with poor outcomes [8, 9].

The differential diagnosis of new‐onset neurological symptoms during induction therapy is broad and includes leukemic infiltration, cerebral hemorrhage or infarction, drug‐induced leukoencephalopathy (particularly from high‐dose cytarabine), meningitis, and other CNS infections [10]. Importantly, newly developed focal neurological deficits during neutrophil recovery should prompt immediate neuroimaging, even when systemic signs of infection appear to be improving. Serious CNS complications may emerge during apparent marrow recovery and can be underestimated if attention is focused only on hematologic improvement.

This case highlights the occurrence of a brain abscess caused by *Bacillus* species following CPX‐351 induction therapy. CPX‐351 is known to induce prolonged neutropenia with relatively low gastrointestinal toxicity compared with conventional “7 + 3” regimens [2]. Although this pharmacologic profile may reduce the risk of gut translocation of Gram‐negative bacilli, prolonged neutropenia may preferentially predispose patients to Gram‐positive bloodstream infections, particularly catheter‐related infections, which can subsequently lead to deep‐seated complications such as a brain abscess. Our patient first developed CRBSI and later presented with a brain abscess during apparent hematologic recovery, highlighting a potential diagnostic pitfall.

Management of brain abscesses in immunocompromised patients generally requires a multimodal and multidisciplinary approach involving neurosurgery, infectious disease expertise, and timely radiologic assessment [11, 12]. Antibiotic therapy alone is often insufficient; surgical drainage or resection is usually necessary for diagnostic confirmation and effective source control [4, 7, 10, 11]. In the present case, vancomycin was replaced with linezolid because of persistent radiological disease and the superior CNS penetration of linezolid, rather than because of microbiological resistance alone. Repeated burr hole drainage followed by endoscopic abscess resection, combined with appropriate antimicrobial therapy, achieved successful infection control and progressive neurological recovery. In summary, clinicians should consider brain abscess as a differential diagnosis in AML patients receiving CPX‐351 who develop new‐onset neurological symptoms. Early recognition, combined with prompt surgical and medical intervention, is crucial for improving outcomes and ensuring successful continuation of leukemia‐directed therapy.

## 4. Conclusion

This case highlights a brain abscess as a rare but serious complication during CPX‐351 treatment for AML–MRC. Clinicians should be aware that focal neurological symptoms may emerge even during apparent hematologic recovery, and prompt neuroimaging with timely multidisciplinary management is essential for favorable outcomes.

## Author Contributions

Treating physicians: Yusuke Yamaga, Yuri Sakuma, and Daimon Siraisi.

Project administration and supervision: Takahiro Nishiyama.

Validation and writing: Yusuke Yamaga.

## Funding

This study received no specific funding.

## Disclosure

All authors have read and approved the final version of the manuscript. Dr. Yusuke Yamaga had full access to all of the data in this study and takes complete responsibility for the integrity of the data and the accuracy of the data analysis.

## Ethics Statement

According to the ethical guidelines for medical and biological research involving human subjects in Japan, the Ichinomiya Municipal Hospital Clinical Research Review Committee does not require ethical approval for a case report.

## Consent

Written informed consent for publication was not obtained. However, this case report has been fully anonymized in accordance with the ICMJE guidelines, and no identifiable personal information is included. The publication of this case was reviewed and approved by the Ethics Committee of Ichinomiya Municipal Hospital.

## Conflicts of Interest

The authors declare no conflicts of interest.

## Data Availability

Data sharing is not applicable to this article as no datasets were generated or analyzed during the current study.
